# Anti-Angiogenesis Maintenance Therapy in Newly Diagnosed and Relapsed Ovarian Cancer: A Meta-analysis of Phase III Randomized Controlled Trials

**DOI:** 10.3389/fphar.2021.726278

**Published:** 2021-11-17

**Authors:** Yizi Wang, Shitai Zhang, Zixuan Song, Ling Ouyang, Yan Li

**Affiliations:** Department of Obstetrics and Gynecology, Shengjing Hospital of China Medical University, Shenyang, China

**Keywords:** anti-angiogenesis, meta-analysis, newly diagnosed ovarian cancer, relapsed ovarian cancer, adverse events

## Abstract

**Aim:** Anti-angiogenesis agents have been added as maintenance therapy in ovarian cancer over the past decade. The aim of this meta-analysis was to analyze the efficacy of anti-angiogenesis therapy in newly diagnosed and relapsed ovarian cancer.

**Methods:** PubMed, Embase, and Cochrane databases were searched for all phase III randomized controlled trials (RCTs) that assessed the efficacy and toxicity of anti-angiogenesis agents in ovarian cancer. Overall survival (OS) and progression-free survival (PFS) were used to evaluate the effectiveness of anti-angiogenesis therapy in ovarian cancer.

**Results:** A total of 6097 patients with newly diagnosed ovarian cancer from 5 phase III RCTs and 2943 patients with relapsed ovarian cancer from 6 phase III RCTs were included in this meta-analysis. The pooled results showed that anti-angiogenesis maintenance therapy significantly improved PFS (hazard ratio [HR], 0.84; 95% confidence interval [CI], 0.76–0.93; *p* = 0.001), but not OS (HR, 0.98; 95% CI, 0.91–1.05; *p* = 0.49) compared with placebo in patients with newly diagnosed ovarian cancer. In patients with relapsed ovarian cancer, the pooled results showed a significant improvement on OS (HR, 0.89; 95% CI, 0.82–0.98; *p* = 0.02) and PFS (HR, 0.61; 95% CI, 0.52–0.72; *p* < 0.001). The pooled results also showed that the anti-angiogenesis agents were associated with an increase in the occurrence of severe hypertension, neutropenia, diarrhea, thrombocytopenia, headache, and bleeding in ovarian cancer. However, infrequent fatal adverse events occurred in the anti-angiogenesis groups.

**Conclusions:** Study results suggest that anti-angiogenesis agents were an effective therapy for newly diagnosed and relapsed ovarian cancer, especially for relapsed ovarian cancer. Anti-angiogenesis agents may be associated with some severe but not fatal adverse events.

**Systematic Review Registration:** https://www.crd.york.ac.uk/prospero/, identifier CRD42021283647

## Introduction

Ovarian cancer is the fifth lethal cancer of all malignancies, and it is the first leading cause of death from gynecologic cancer (Siegel et al., 2021). Despite standard treatment with surgery combined with platinum-based chemotherapy, the majority of patients relapse within 5 years (Bartoletti et al., 2020).

Angiogenesis plays a key role in the growth and progression of malignant tumors through a complex process (Coleman et al., 2013; Monk et al., 2016a), which is distinctly related to vascular endothelial growth factor (VEGF) and the angiopoietin-Tie2 receptor complex (Gavalas et al., 2013). The VEGF pathway has been widely studied in carcinogenesis, with the agents bevacizumab, cediranib, pazopanib, and nintedanib having been proved to treat solid tumors through inhibition of the VEGF pathway (Ferrara and Adamis, 2016; Jayson et al., 2016). Angiopoietin 1 (Ang1) and angiopoietin 2 (Ang2) interact with the Tie2 receptor, increasing blood vessel density (Falcón et al., 2009). Trebananib has been proved to target this pathway by neutralizing Ang1 and Ang2 and then preventing their interaction with the Tie2 receptor (Coxon et al., 2010).

Several anti-angiogenic agents have been researched in ovarian cancer. Bevacizumab was proved to be effective in improving the progression-free survival (PFS) of patients with ovarian cancer in the first phase III randomized controlled trial (RCT) (Perren et al., 2011). Then, more RCTs were designed to evaluate the effect of anti-angiogenesis therapy in ovarian cancer. However, the prognoses of patients with newly diagnosed or relapsed ovarian cancer reported by these RCTs are not consistent.

From the results of phase III RCTs of anti-angiogenesis agents that have reported the final PFS, overall survival (OS), and safety analysis, we performed a meta-analysis aiming to assess the efficacy of anti-angiogenesis maintenance therapy for patients with newly diagnosed or relapsed ovarian cancer.

## Methods

### Literature Search

In this meta-analysis performed following the Preferred Reporting Items for Systematic Reviews and Meta-Analyses (PRISMA) guidelines, a search was carried out of the Embase, PubMed, Web of Science, and Cochrane databases. The meta-analysis included all publications that reported the results of phase III RCTs before January 30, 2021. The comprehensive search strings were “ovarian neoplasm,” “ovarian cancer,” “ovarian malignancy,” “ovarian carcinoma”, and associated terms; as well as “anti-angiogenesis,” “bevacizumab,” “nintedanib,” “pazopanib,” “nintedanib,” “cediranib,” “trebananib.” Searches were performed without any restriction on language or publication year. Manual searches for other potential studies were conducted using the reference lists of the selected studies.

### Eligibility Criteria

Phase III RCTs were included if they met the following inclusion criteria in accordance with PICOS (population, intervention, comparison, outcomes and study design) guidelines: 1) patients with newly diagnosed or relapsed ovarian cancer; 2) patients administered anti-angiogenesis agents as a maintenance treatment; 3) the comparison made was anti-angiogenesis agent vs placebo; 4) OS and PFS were compared between the group receiving anti-angiogenesis treatment and the placebo group; and 5) the study design included phase III RCTs.

Studies were excluded if they were non-RCTs, phase I or II RCTs, review articles, case reports, editorials, letters, or conference abstracts. We also excluded any studies that lacked OS or PFS and those with patient populations that were duplicated in another study.

### Selection and Extraction

Two of the authors (Wang and Zhang) independently identified and selected the studies according with the inclusion and exclusion criteria. The following data elements were extracted from each trial: first author, publication year, clinical trial acronym, medication, disease setting, study period, follow-up time, total patients, PFS, and OS. The Cochrane Collaboration tool was used to assess the risk of bias in the trial (Higgins et al., 2011). Disagreements between the 2 reviewers were identified and resolved by referral to a third author (Song) and by consensus.

### Statistical Analysis

We extracted hazard ratios (HRs) and 95% confidence intervals (CIs) of the OS and PFS from all the included trials. We also calculate the odds ratio (OR) to determine the patients’ severe toxicity profile (G3-G4 toxicity) of the anti-angiogenesis agents administered. The meta-analysis was performed using Stata software, version 12.0 (StataCorp) and Review Manager 5.3. Pooled HRs were obtained using random-effects models to reduce the heterogeneity between studies (DerSimonian and Laird, 1986). Heterogeneity between studies was evaluated using the χ^2^ test and *I* ^2^ statistic, and *I* ^2^ values of less than 25%, 25–75%, and greater than 75% were considered low, moderate, and high heterogeneity, respectively (Higgins et al., 2003). The robustness of the main findings were assessed using sensitivity analyses (Copas and Shi, 2000). We also performed subgroup analyses to confirm if the heterogeneity results were moderate or higher. Funnel plots with Begg’s and Egger’s regressions were used to visually examine the effect of publication bias (Begg and Mazumdar, 1994; Egger et al., 1997). A *p* value less than 0.05 was considered significant, and all *p* values were 2-sided.

## Results

### Study Selection

**FIGURE 1:**
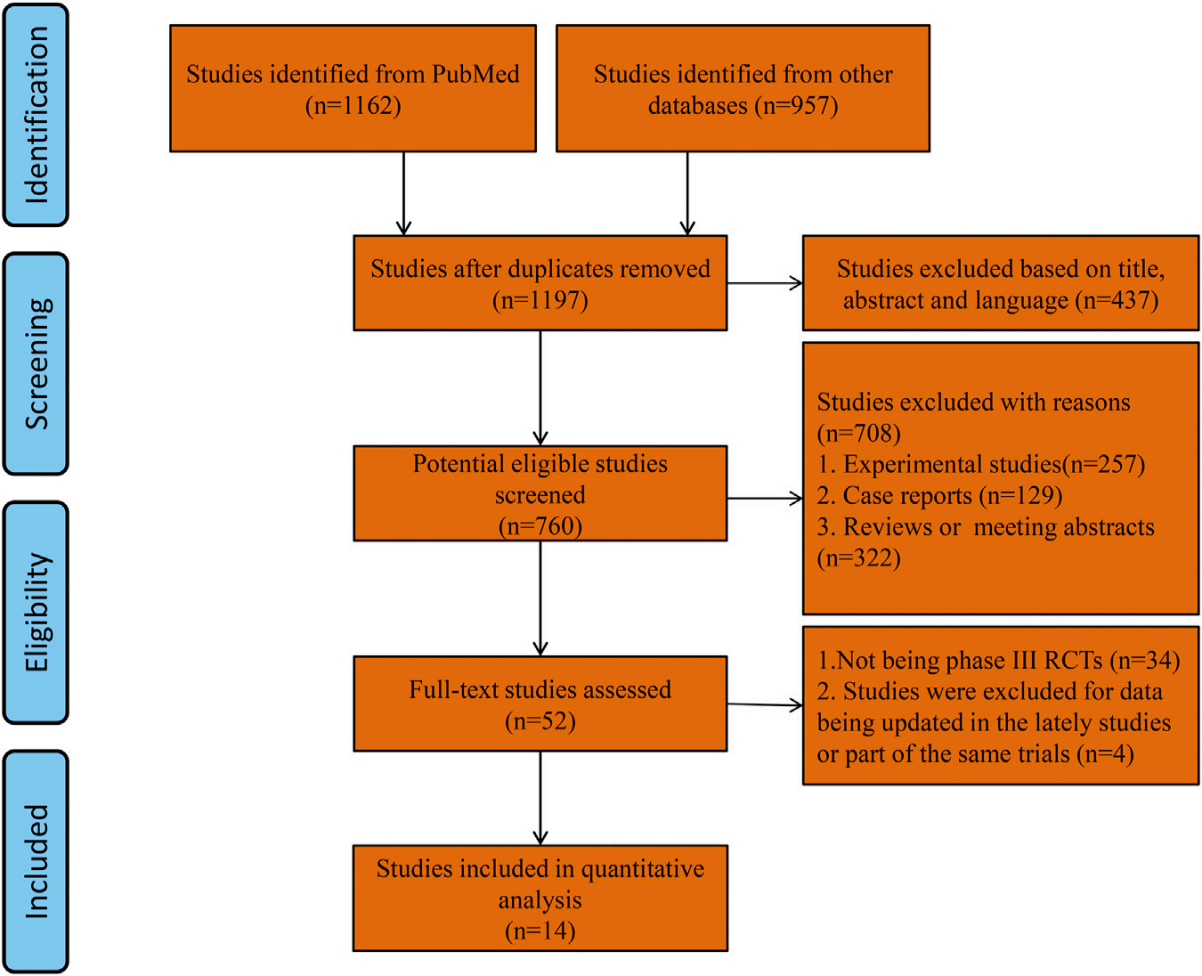
Flow diagram of trial selection.

After the initial comprehensive search, a total of 1197 studies were retrieved. Using the abstracts or titles of the articles during the preliminary screening, 52 full-text papers were further scrutinized. Thirty-four publications were excluded because they were not phase III RCTs. Three studies (Perren et al., 2011; du Bois et al., 2016; Monk et al., 2014) were excluded because the survival outcome data were updated in the lately three studies with the same trials. One study was excluded because the anti-angiogenesis treatment trial was conducted in the subgroup of a rare gynecologic ovarian cancer (Gore et al., 2019). Eventually, 11 phase III RCTs were selected as they fulfilled all the study inclusion criteria. A total of 6097 patients with newly diagnosed ovarian cancer from 5 phase III RCTs and 2943 patients with relapsed ovarian cancer from 6 phase III RCTs were included in this meta-analysis. As the OS and PFS data of 3 trials were reported separately in different publications, we included 14 publications in our study (Figure 1). Five RCTs reported the results of patients with newly diagnosed ovarian cancer (anti-angiogenesis group = 3,448; placebo group = 2649; total = 6097 patients): ICON7, GOG-0218, AGO-OVAR16, AGO-OVAR 12, TRINOVA-3 (Ray-Coquard et al., 2020; Vergote et al., 2019a; Vergote et al., 2019b; du Bois et al., 2014; Tewari et al., 2019; Burger et al., 2011; Oza et al., 2015). The other 6 RCTs reported the results of patients with recurrent ovarian cancer (anti-angiogenesis group = 1497; placebo group = 1446; total = 2943 patients): OCEANS, ICON6, TRINOVA-1, TRINOVA-2, GOG-0213, AURELIA (Aghajanian et al., 2012; Pujade-Lauraine et al., 2014; Aghajanian et al., 2015; Monk et al., 2016b; Ledermann et al., 2016; Coleman et al., 2017; Marth et al., 2017). The risk of bias was globally low that was illustrated using “Risk of bias graph” (Supplementary Figure S1). Characteristics of the individual studies and enrolled population are summarized in Table 1.

**TABLE 1:** Characteristics of the individual studies and enrolled population.

Study	Trial acronym	Disease setting	Medication	Study period	Follow-up (median months)	Number of patients enrolled		
						Total	Trial	Control
Vergote 2019	TRINOVA-3	newly diagnosed	trebananib	2012–2014	27.4	1015	678	337
Ray-Coquard 2020	AGO-OVAR 12	newly diagnosed	nintedanib	2009–2011	60.9	1366	911	455
Vergote 2019	AGO-OVAR16	newly diagnosed	pazopanib	2009–2010	NA	940	472	468
du Bois 2014	AGO-OVAR16	newly diagnosed	pazopanib	2009–2010	24.3	940	472	468
Oza 2015	ICON7	newly diagnosed	bevacizumab	2006–2009	48.9	1528	764	764
Tewari 2019	GOG-0218	newly diagnosed	bevacizumab	2005–2009	102.9	1248	623	625
Burger 2011	GOG-0218	newly diagnosed	bevacizumab	2005–2009	17.4	1248	623	625
Monk 2016	TRINOVA-1	relapse	trebananib	2010–2012	18	919	461	458
Aghajanian 2015	OCEANS	relapse	bevacizumab	2007–2010	58.2	484	242	242
Aghajanian 2012	OCEANS	relapse	bevacizumab	2007–2010	24	484	242	242
Coleman 2017	GOG-0213	relapse	bevacizumab	2007–2011	49.6	674	337	337
Pujade-Lauraine 2014	AURELIA	relapse	bevacizumab	2009–2011	13	361	179	182
Ledermann 2016	ICON6	relapse	cediranib	2007–2011	19.5	282	164	118
Marth 2017	TRINOVA-2	relapse	Trebananib	2011–2013	12.4	223	114	109

NA, not available.

### Anti-Angiogenesis Maintenance Treatment in Newly Diagnosed Ovarian Cancer

**FIGURE 2:**
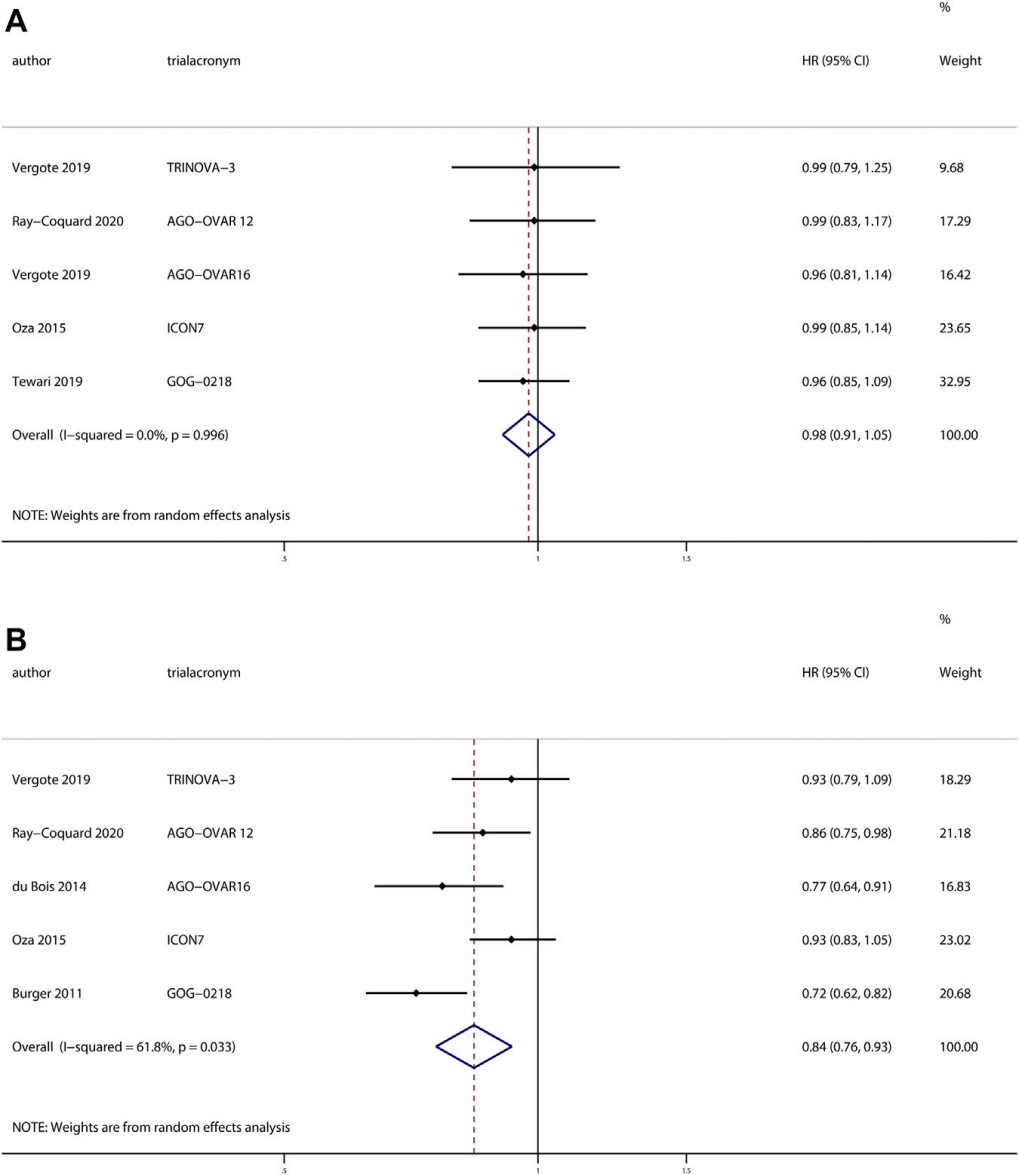
Anti-angiogenesis maintenance treatment vs placebo in newly diagnosed ovarian cancer. **(A)** Overall survival (OS). **(B)** Progression-free survival (PFS).

Five trials (Ray-Coquard et al., 2020; Vergote et al., 2019a; Vergote et al., 2019b; du Bois et al., 2014; Tewari et al., 2019; Burger et al., 2011; Oza et al., 2015) reported the effects of anti-angiogenesis maintenance therapy in patients with newly diagnosed ovarian cancer. A total of 3,448 patients received chemotherapy followed by anti-angiogenesis maintenance therapy, whereas 2649 patients received maintenance therapy with placebo. There was no difference in OS between the two groups (HR, 0.98; 95% CI, 0.91–1.05; *p* = 0.49). However, the pooled results revealed a significant improvement in PFS for patients receiving anti-angiogenesis maintenance therapy compared with placebo (HR, 0.84; 95% CI, 0.76–0.93; *p* = 0.001) (Figure 2).

No heterogeneity was observed in OS (χ^2^ = 0.18, *p* > 0.99, I^2^ = 0%), but a moderate degree of heterogeneity existed in terms of PFS (χ^2^ = 10.48, *p* = 0.03, I^2^ = 61.8%), sensitivity analyses revealed the same results when we excluded individual studies one by one (Supplementary Figure S2).

Funnel plots showed little evidence of asymmetry in those assessing OS (Begg’s test, *p* > 0.99; Egger’s test, *p* = 0.42) and PFS (Begg’s test, *p* = 0.46; Egger’s test, *p* = 0.57), which revealed the absence of publication bias (Supplementary Figure S3).

Investigators of the ICON7 study defined the high-risk tumors (International Federation of Gynecology and Obstetrics [FIGO] stage IV, or FIGO stage III if residual tumor after debulking surgery was >1.0 cm), and investigators of three trials (Ray-Coquard et al., 2020; du Bois et al., 2014; Oza et al., 2015) reported the effects of anti-angiogenesis therapy in high-risk and non-high-risk patients. We evaluated these data into subgroup analyses. The results revealed a significant improvement in PFS for patients receiving anti-angiogenesis maintenance treatment (HR, 0.80; 95% CI, 0.65–0.97; *p* = 0.02), but no significant difference in OS (HR, 0.94; 95% CI, 0.65–1.36; *p* = 0.74) compared with placebo in high-risk patients. However, in the complementary subgroup of non-high-risk patients, there were no significant differences in OS (HR, 1.02; 95% CI, 0.80–1.29; *p* = 0.90) or PFS (HR, 0.87; 95% CI, 0.72–1.06; *p* = 0.17).

**FIGURE 3:**
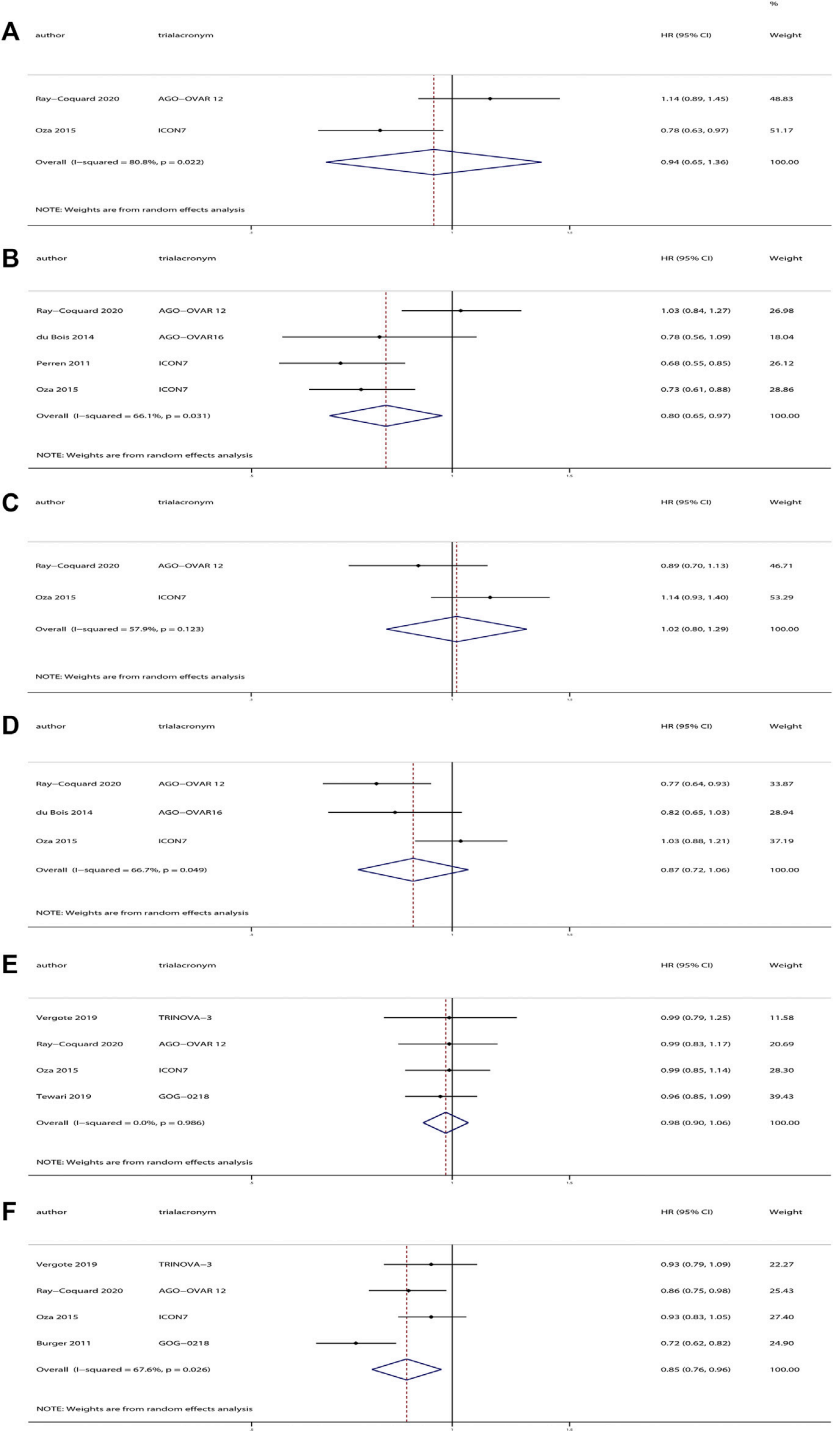
Subgroup analyses of anti-angiogenesis treatment vs placebo in newly diagnosed ovarian cancer. **(A)** Overall survival (OS) of patients with high-risk tumors. **(B)** Progression-free survival (PFS) of patients with high-risk tumors. **(C)** OS of patients with non-high-risk tumors. **(D)** PFS of patients with non-high-risk tumors. **(E)** OS of patients who received anti-angiogenesis treatment in combination with chemotherapy followed by anti-angiogenesis maintenance treatment vs. placebo. **(F)** PFS of patients who received anti-angiogenesis treatment in combination with chemotherapy followed by anti-angiogenesis maintenance treatment vs. placebo.

Among these trials, the AGO-OVAR16 trial evaluated 472 patients who received anti-angiogenesis maintenance treatment after completion of standard first-line chemotherapy, whereas the other four trials contained a total of 2976 patients who received anti-angiogenesis agents in combination with chemotherapy followed by anti-angiogenesis maintenance treatment. We pooled the results of the four trials and found a significant improvement in PFS for patients receiving anti-angiogenesis maintenance therapy (HR, 0.85; 95% CI, 0.76–0.96; *p* = 0.01), but no significant difference in OS (HR, 0.98; 95% CI, 0.90–1.06; *p* = 0.58) compared with placebo. The results of the subgroup analyses are depicted in Figure 3.

### Anti-Angiogenesis Maintenance Treatment in Relapsed Ovarian Cancer

**FIGURE 4:**
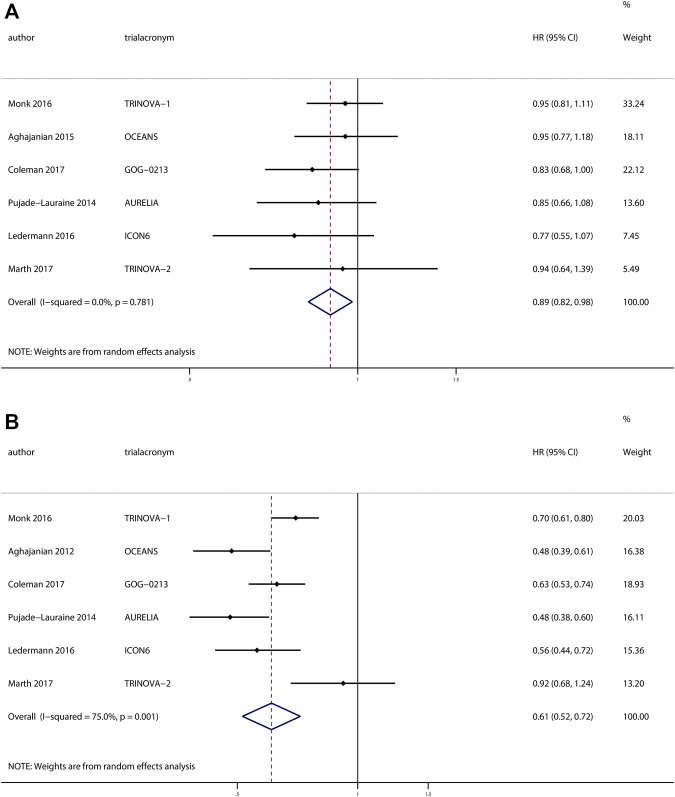
Anti-angiogenesis maintenance treatment vs placebo in relapsed ovarian cancer. **(A)** Overall survival (OS). **(B)** Progression-free survival (PFS).

The investigators of six trials with 2943 patients reported the effects of anti-angiogenesis maintenance therapy for relapsed ovarian cancer. A total of 1497 patients with recurrent ovarian cancer received chemotherapy followed by anti-angiogenesis maintenance therapy, whereas 1446 women received placebo. The pooled results revealed a significant improvement in OS (HR, 0.89; 95% CI, 0.82–0.98; *p* = 0.02) and PFS (HR, 0.61; 95% CI, 0.52–0.72; *p* < 0.001) (Figure 4) between the two groups.

No heterogeneity was observed in OS (χ^2^ = 2.47, *p* = 0.78, I^2^ = 0%), but a moderate degree of heterogeneity existed in terms of PFS (χ^2^ = 19.99, *p* = 0.001, I^2^ = 75.0%). We performed a sensitivity analysis based on the PFS and obtained the same results when we excluded studies one by one (Supplementary Figure S4).

Visual inspection of the funnel plots shows little evidence of asymmetry in plots assessing OS (Begg’s test, *p* = 0.45; Egger’s test, *p* = 0.43) and PFS (Begg’s test, *p* > 0.99; Egger’s test, *p* = 0.67), which revealed the absence of publication bias (Supplementary Figure S5).

Among these trials, patients from three trials (Aghajanian et al., 2012; Ledermann et al., 2016; Coleman et al., 2017) received platinum-based therapy followed by anti-angiogenesis for maintenance, whereas patients from the other three trials (Pujade-Lauraine et al., 2014; Monk et al., 2016b; Marth et al., 2017) received non–platinum-based therapy followed by anti-angiogenesis therapy for maintenance. Hence, we performed subgroup analyses based on platinum-based therapy or non–platinum-based therapy. With respect to OS, we observed a significant improvement in OS for patients receiving platinum-based therapy followed by anti-angiogenesis treatment for maintenance (HR, 0.86; 95% CI, 0.76–0.98; *p* = 0.03) compared with placebo, but no significant difference in OS for patients receiving non–platinum-based therapy followed by anti-angiogenesis treatment for maintenance (HR, 0.92; 95% CI, 0.81–1.05; *p* = 0.20) compared with placebo. At the same time, we found both an improved PFS (HR, 0.56; 95% CI, 0.48–0.66; *p* < 0.001) for patients receiving platinum-based therapy followed by anti-angiogenesis for maintenance compared with placebo and an improved PFS (HR, 0.67; 95% CI, 0.49–0.92; *p* = 0.01) for patients receiving non–platinum-based therapy followed by anti-angiogenesis for maintenance therapy compared with placebo.

**FIGURE 5:**
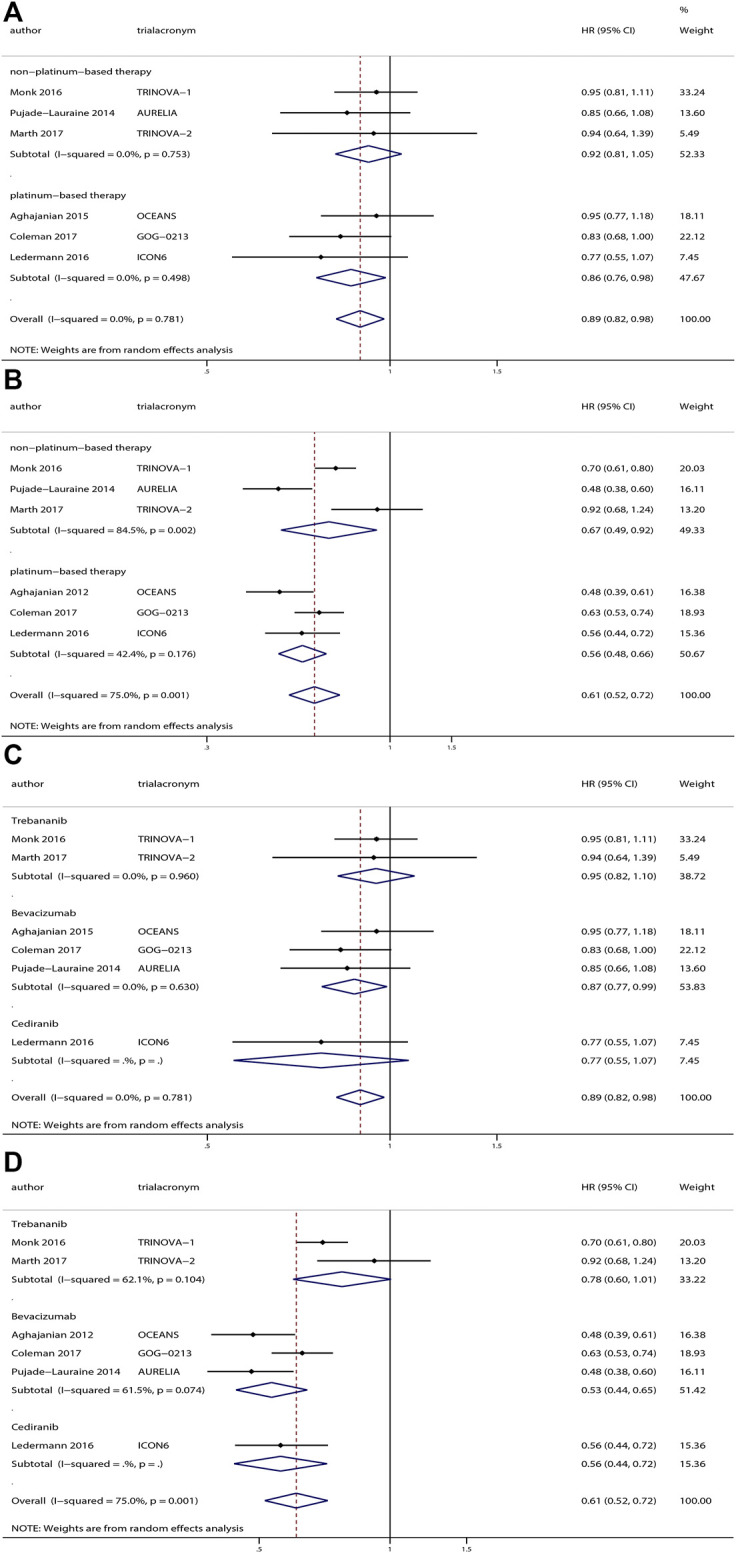
Subgroup analyses of anti-angiogenesis treatment vs placebo in relapsed ovarian cancer. Subgroup analyses based on platinum-based therapy or non–platinum-based therapy: **(A)** overall survival (OS) and **(B)** progression-free survival (PFS). Subgroup based on drugs: **(C)** OS and (D) PFS.

Subgroups based on specific medications were also analyzed. Three trials (Aghajanian et al., 2012; Pujade-Lauraine et al., 2014; Coleman et al., 2017) researched bevacizumab, and the pooled results revealed a significant improvement in OS (HR, 0.87; 95% CI, 0.77–0.99; *p* = 0.03) and PFS (HR, 0.53; 95% CI, 0.44–0.65; *p* < 0.001) compared with placebo. However, trebananib and cediranib did not demonstrate superiority compared with placebo. The subgroup analysis results are depicted in Figure 5.

### Safety of Anti-Angiogenesis Maintenance Therapy

All the included trials investigated the adverse events of anti-angiogenesis therapy in patients. The most common adverse events were hypertension, diarrhea, headache, and proteinuria. In addition, we pooled and analyzed the results from individual trials regarding the severe adverse events (Grade ≥3).

Of the five trials that evaluated patients with newly diagnosed ovarian cancer, we analyzed the results of severe adverse events. The pooled results revealed that there were significant differences in terms of severe hypertension, neutropenia, diarrhea, thrombocytopenia, headache, proteinuria, hypokalemia, and bleeding in patients with newly diagnosed ovarian cancer who received anti-angiogenesis therapy compared with placebo (Supplementary Figure S6).

The investigators of six trials reported adverse events of anti-angiogenesis maintenance therapy in patients with relapsed ovarian cancer, and we analyzed the results of severe adverse events. We observed significant differences in severe hypertension, headache, proteinuria, bleeding, localized edema, and ascites in patients with relapsed ovarian cancer patients who received anti-angiogenesis therapy compared with placebo (Supplementary Figure S7).

Regarding to the occurrence of severe nausea, vomiting, fatigue, abdominal pain, and asthenia, we observed no significant differences between the anti-angiogenesis and placebo groups regardless of whether or not the ovarian cancer was newly diagnosed or relapsed based on the pooled analyses.

## Discussion

Ovarian cancer is usually detected during the advanced stages due to the atypical symptoms seen during earlier stages; it has the worst prognosis of all the gynecologic malignancies (Dinkelspiel et al., 2015). Almost all patients with advanced ovarian cancer will undergo relapse, and then chemoresistance commonly occurs (Roane et al., 2019). In previous years, targeted agents have been used to treat advanced ovarian cancer and have proved to be effective. These include the poly (ADP-ribose) polymerase (PARP) inhibitors (Tomao et al., 2019; Ruscito et al., 2020). At the same time, anti-angiogenesis maintenance therapy has been researched in some phase III trials, however, the reported results were not consistent.

Our meta-analysis included 11 trials enrolling a total of 9,040 patients with ovarian cancer. Five of these trials reported anti-angiogenesis maintenance therapy in newly diagnosed ovarian, and the other six trials researched anti-angiogenesis in relapsed ovarian cancer.

Each of the five trials we included reported PFS and the final OS results. We found that all of the trials reported no significant differences based on OS in patients with newly diagnosed ovarian cancer, although GOG-0218, AGO-OVAR 12, and AGO-OVAR16 observed that bevacizumab, nintedanib, or pazopanib, respectively, could improve the PFS. However, ICON7 and TRINOVA-3 did not observe an improved PFS. The ICON7 study performed a subgroup analysis of patients with stage IIIB to stage IV disease and found an improvement in PFS irrespective of residual cancer or stage status and an improved OS in high-risk patients (Oza et al., 2015). Trebananib was the targeted drug in newly diagnosed ovarian cancer in the TRINOVA-3 study, the results of which showed that it did not improve OS or PFS compared with placebo. Our pooled results indicated an improved PFS in patients with newly diagnosed ovarian cancer who received anti-angiogenesis maintenance therapy compared with placebo. However, there was no significant difference in OS in patients with newly diagnosed ovarian cancer between the anti-angiogenesis and placebo groups. In addition, we conducted subgroup analyses based on high-risk or non-high-risk tumours. We obtained similar results that anti-angiogenesis maintenance therapy could improve PFS but not OS in high-risk patients. However, in the subgroup analysis of non-high-risk patients, there were no significant differences in OS or PFS between the anti-angiogenesis and placebo groups. We also pooled the results of trials that combined anti-angiogenesis with first-line chemotherapy followed by anti-angiogenesis maintenance therapy, which revealed a significant improvement in PFS but not OS for patients receiving anti-angiogenesis maintenance compared with placebo.

From the previously mentioned results, we found that, although anti-angiogenesis treatment could improve PFS, patients with newly diagnosed ovarian cancer did not benefit from anti-angiogenesis treatment in terms of OS. The most likely explanation for this was that OS might be affected by many factors. Yang et al. found that patients with long-term survival likely have higher mutation frequency, such as BRCA mutation (Yang et al., 2018). Iwase et al. found that OS from the time of the first recurrence might be not associated with the initial FIGO stage because the OS could be prolonged in the patients with relapsed ovarian cancer who were sensitive to chemotherapy, regardless of the original stage (Iwase et al., 2015). Some authors have demonstrated that primary complete or optimal cytoreductive surgery (Chan et al., 2003; Kaern et al., 2005), preoperative disease burden (Horowitz et al., 2015; Hamilton et al., 2018; Javellana et al., 2019), multiple lines of prior therapy (Pignata et al., 2017), and disease-free interval are independent prognostic factors for OS (Kajiyama et al., 2012; Hoppenot et al., 2018). Unexpected crossover, inconsistent treatment, and survival time after progression may not reflect the real effect of new drugs on OS (Tewari et al., 2019). However, we couldn’t balance factors such as the patients’ genetic variant status, therapy after recurrence, or response to the chemotherapy when we performed the meta-analysis.

Although the pooled results didn’t support the role of anti-angiogenesis therapy in newly diagnosed ovarian cancer, OS has improved in the past several decades. The 5-year relative survival rate for ovarian cancer was 36% between 1975 and 1997. This rate has increased year by year, and it improved significantly to 49% between 2010 and 2016 (Siegel et al., 20152015). This improvement reflects the advances in treatment of ovarian cancer, including improved surgical interventions and multiple lines of therapy for recurrence. The US Food and Drug Administration (FDA) has reported that novel agents are approved depending on the PFS combined with comprehensive factors of quality of life and toxicities (Herzog et al., 2017). As an improved PFS was observed in our meta-analysis, this may be a possible reason for application of anti-angiogenesis treatment in these patients.

When we performed analyses of patients with relapsed ovarian cancer, we found anti-angiogenesis maintenance therapy could improve both OS and PFS compared with placebo. All the trials reported a significantly improved PFS in patients who received anti-angiogenesis maintenance therapy except for the TRINOVA-2 trial. Although the individual trials didn’t observe an improved OS, we found a significant improvement in OS when we pooled all the individual results. At the same time, there was no heterogeneity or publication bias observed in OS. The most likely explanation is that when increasing the sample size, the efficiency was improved which reduced the possibility of a false negative.

In the subgroup analyses, OCEANS, GOG-0213, and ICON6 administered anti-angiogenesis treatment to patients who received platinum-based therapy, whereas patients in the TRINOVA-1, AURELIA, and TRINOVA-2 trials received non–platinum-based therapy. The pooled results revealed that anti-angiogenesis maintenance therapy could improve PFS both in patients who received platinum-based and non–platinum-based therapy. However, an improved OS was observed in patients who received platinum-based therapy plus anti-angiogenesis maintenance therapy but not in patients who received non–platinum-based therapy. The TRINOVA-1 and TRINOVA-2 trials evaluated trebananib plus paclitaxel or pegylated liposomal doxorubicin, respectively. An improved PFS was observed in TRINOVA-1 but not in TRINOVA-2. The ICON6 trial found an improved PFS in patients who received platinum-based therapy followed by treatment with cediranib. The previous review included two RCTs of bevacizumab use in relapsed ovarian cancer, and found a statistically significant improvement in PFS but not in OS (Rossi et al., 2017). In our meta-analysis, three trials (Aghajanian et al., 2012; Pujade-Lauraine et al., 2014; Coleman et al., 2017)researched bevacizumab as maintenance therapy, and we pooled the results which revealed a significant improvement in both PFS and OS in patients with relapsed ovarian cancer.

Stark et al. reported no decreased health-related quality of life in patients with relapsed ovarian cancer who received anti-angiogenesis therapy (Stark et al., 2017). Higher frequency of serious hypertension occurred in older patients who received bevacizumab therapy (Sorio et al., 2017). In a meta-analysis of bevacizumab treatment for advanced or metastatic breast cancer, the quality of life was similar between the bevacizumab and control groups (Miyashita et al., 2020).

All the trials we included compared the adverse events between anti-angiogenesis treatment and placebo. In the ICON7, OCEANS, GOG-0213, and AURELIA trials, a higher frequency of severe adverse events occurred in patients who received bevacizumab. But the occurrence rate of fatal adverse events was not significant difference between bevacizumab and placebo groups. And in the GOG-0218 trial, there were no significant differences in the occurrence rate of fatal adverse events between the bevacizumab group (2.3%) and the control group (1.0%). In the TRINOVA-1, TRINOVA-2, and TRINOVA-3 trials, the occurrence rate of severe adverse events was similar in the trebananib and placebo group. In the AGO-OVAR 12 trial, more severe adverse events were observed in patients who received nintedanib (81%) than in those who received placebo (67%). However, the occurrence of fatal adverse events was similar in the two groups (3 vs. 4%, respectively). In the AGO-OVAR16 trial, some severe adverse events were more frequently observed in the pazopanib group, such as hypertension, neutropenia, diarrhea, fatigue, and thrombocytopenia. However, the occurrence rate of fatal adverse events was not significant. In the ICON6 trial, the incidence of drug discontinuation was higher in the cediranib group. Most trials reported a grade of greater than or equal to 3 adverse events including hypertension, neutropenia, nausea, fatigue, diarrhea, thrombocytopenia, headache, proteinuria, and dyspnea. We pooled the results and found that severe hypertension, neutropenia, diarrhea, thrombocytopenia, headache, proteinuria, hypokalemia, and bleeding were more frequently observed in patients with newly diagnosed ovarian cancer and that severe hypertension, headache, proteinuria, bleeding, localized edema, and ascites were more likely to occur in patients with relapsed ovarian cancer who received anti-angiogenesis treatment compared with placebo. However, all the trials reported infrequent fatal adverse events in both groups.

To the best of our knowledge, this meta-analysis was the first to assess the effects of anti-angiogenesis maintenance therapy in both patients with newly diagnosed and relapsed ovarian cancer. All the studies included in the meta-analysis were well-designed, high-quality, phase III RCTs.

Inevitably, there are some limitations in our meta-analysis. First, potential heterogeneity existed among the individual studies for the inconsistent tumor characteristics of the enrolled population, such as preoperative disease burden, tumor stage, residual tumor after primary surgery, multiple lines of prior therapy, or disease-free interval; these characteristics were not consistent among the different trials. Because the number of studies was limited, we could not perform stratified pooled analyses for each characteristic. Second, the patients in different trials were administered different anti-angiogenesis drugs. We couldn’t pool the results of studies involving the same drugs due to the limited numbers of trials, except for bevacizumab in relapsed ovarian cancer. Third, the majority of included trials reported OS, which was defined as death regardless of cause. The disease-specific OS is more persuasive, however, we could not obtain this from the included trials. Finally, the number of the trials performed on anti-angiogenesis maintenance therapy in ovarian cancer was limited, and more high-quality phase III RCTs are needed to confirm or update our conclusion. Due to the conflicting results of PFS and OS, future RCTs would be better designed to unify the tumor characteristics of ovarian cancer patients, such as histopathologic type and residual tumors, in an attempt to find the best indication for anti-angiogenesis use.

## Conclusion

The results of our study suggest that anti-angiogenesis maintenance therapy may be associated with improved PFS and OS in patients with relapsed ovarian cancer. However, in patients with newly diagnosed ovarian cancer, anti-angiogenesis therapy may be associated with improved PFS, but not OS. Infrequent fatal adverse events occurred in the anti-angiogenesis and placebo groups.

## Data Availability

The original contributions presented in the study are included in the article/Supplementary Material, further inquiries can be directed to the corresponding author.

## Supplementary Material

Supplementary Figure 1 Risk of bias graph. **(A)** Newly diagnosed ovarian cancer. **(B)** Relapsed ovarian cancer.

Supplementary Figure 2 Sensitivity analyses of the pooled analysis of anti-angiogenesis treatment vs placebo in newly diagnosed ovarian cancer in terms of progression-free survival (PFS).

Supplementary Figure 3 Publication bias for overall survival (OS) analysis of anti-angiogenesis treatment vs placebo in newly diagnosed ovarian cancer. **(A)** Begg’s test. **(B)** Egger’s test. Publication bias for progression-free survival (PFS) analysis. **(C)** Begg’s test. **(D)** Egger’s test.

Supplementary Figure 4 Sensitivity analyses of the pooled analysis of anti-angiogenesis treatment vs. placebo in relapsed ovarian cancer in terms of progression-free survival (PFS).

Supplementary Figure 5 Publication bias for overall survival (OS) analysis of anti-angiogenesis treatment vs placebo in relapsed ovarian cancer. **(A)** Begg’s test. **(B)** Egger’s test. Publication bias for progression-free survival (PFS) analysis. **(C)** Begg’s test. **(D)** Egger’s test.

Supplementary Figure 6 Severe adverse events of anti-angiogenesis maintenance treatment vs. placebo in newly diagnosed ovarian cancer.

Supplementary Figure 7 Severe adverse events of anti-angiogenesis maintenance treatment vs. placebo in relapsed ovarian cancer.
