# Supplementary figures and images for: Anti-Angiogenesis Maintenance Therapy in Newly Diagnosed and Relapsed Ovarian Cancer: A Meta-analysis of Phase III Randomized Controlled Trials
Source: Front Pharmacol. 2021 Nov 17;12:726278. doi: 10.3389/fphar.2021.726278 (PMC8636101; doi:10.3389/fphar.2021.726278)

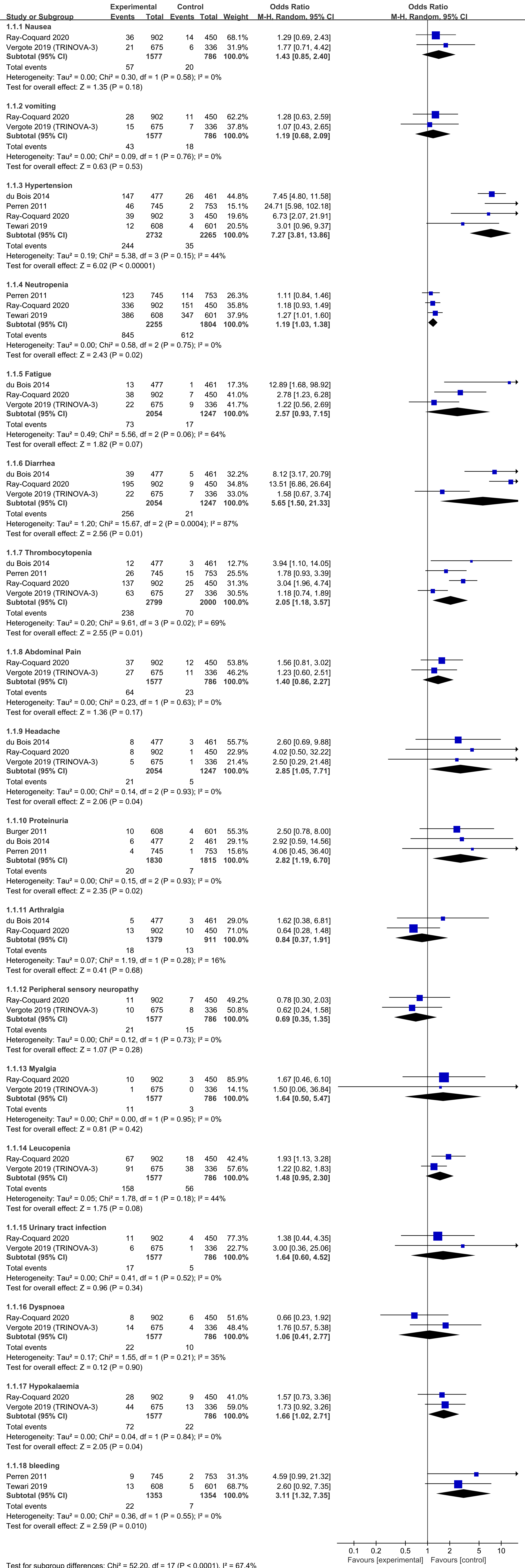

Supplement: Supplementary file 1 [file Image6.TIF]

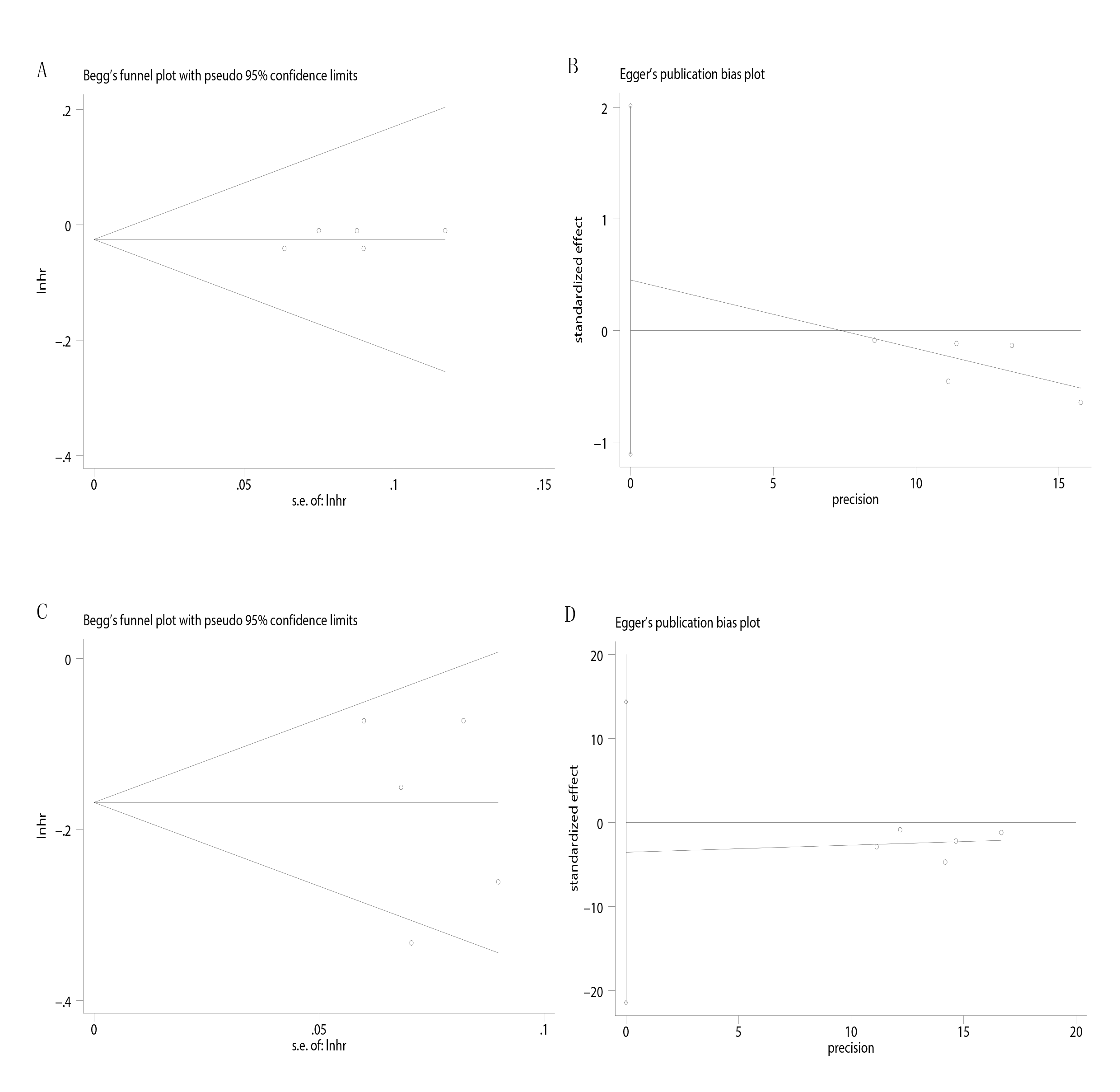

Supplement: Supplementary file 2 [file Image3.TIF]

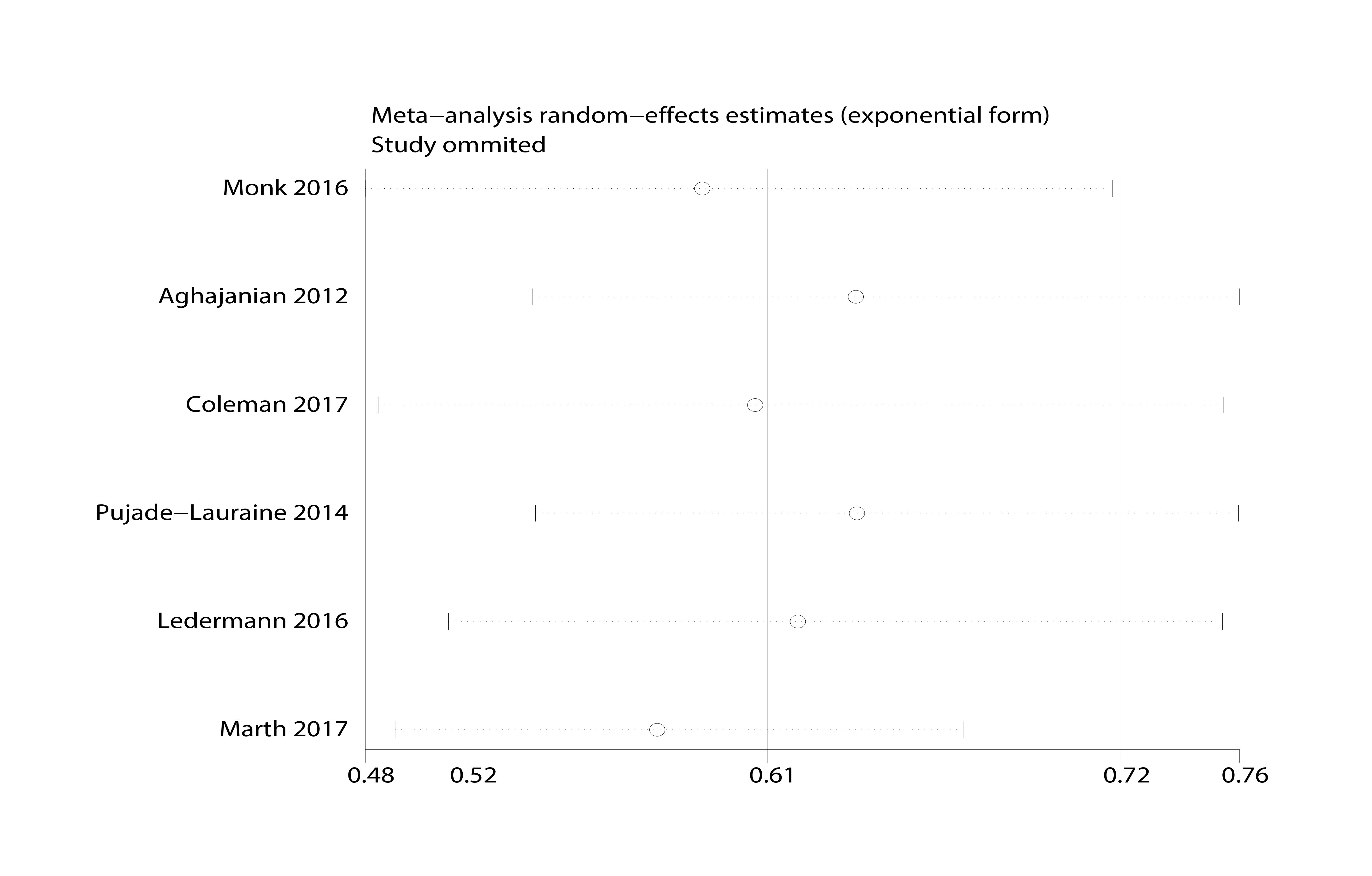

Supplement: Supplementary file 3 [file Image4.TIF]

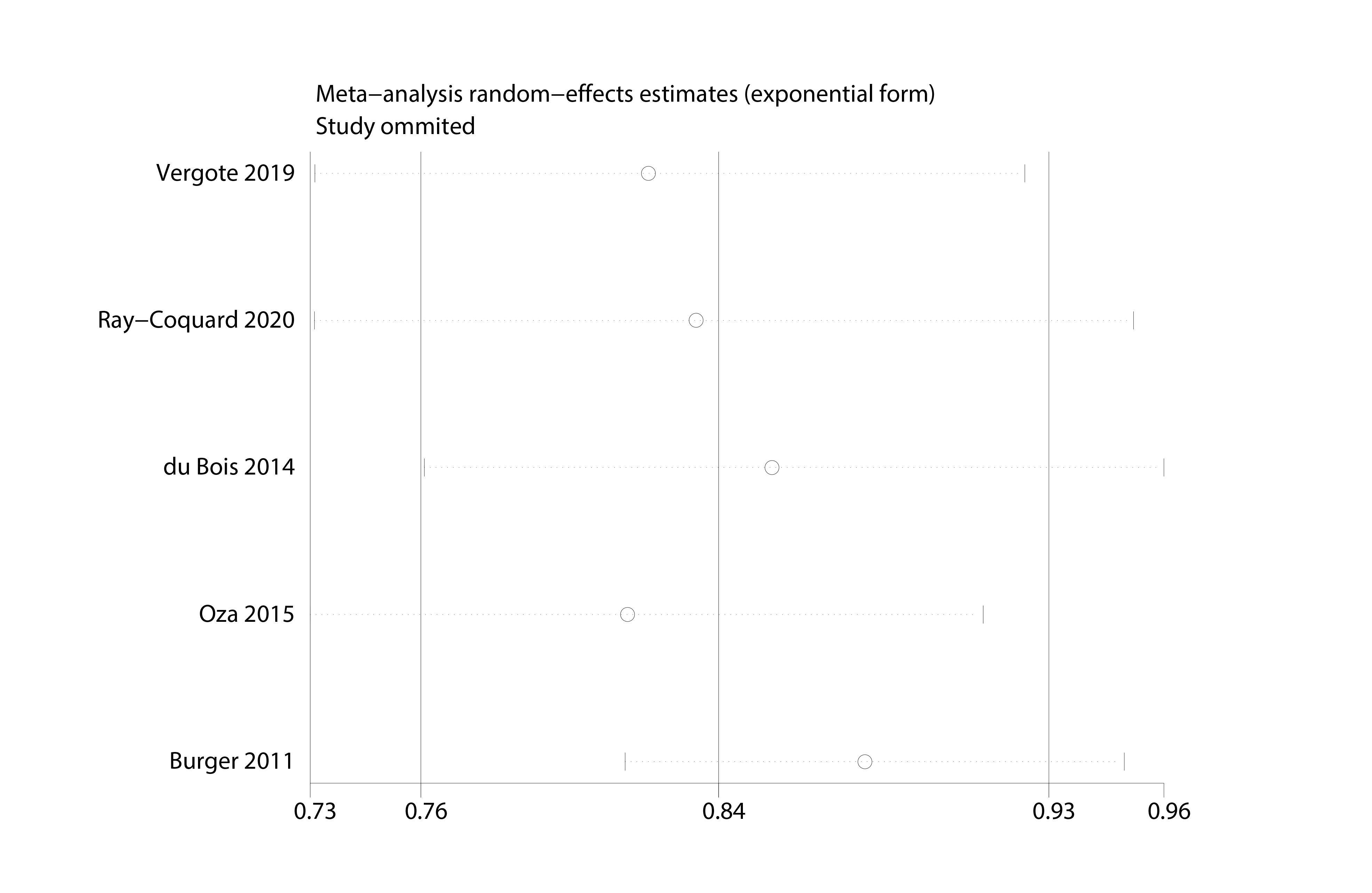

Supplement: Supplementary file 4 [file Image2.TIF]

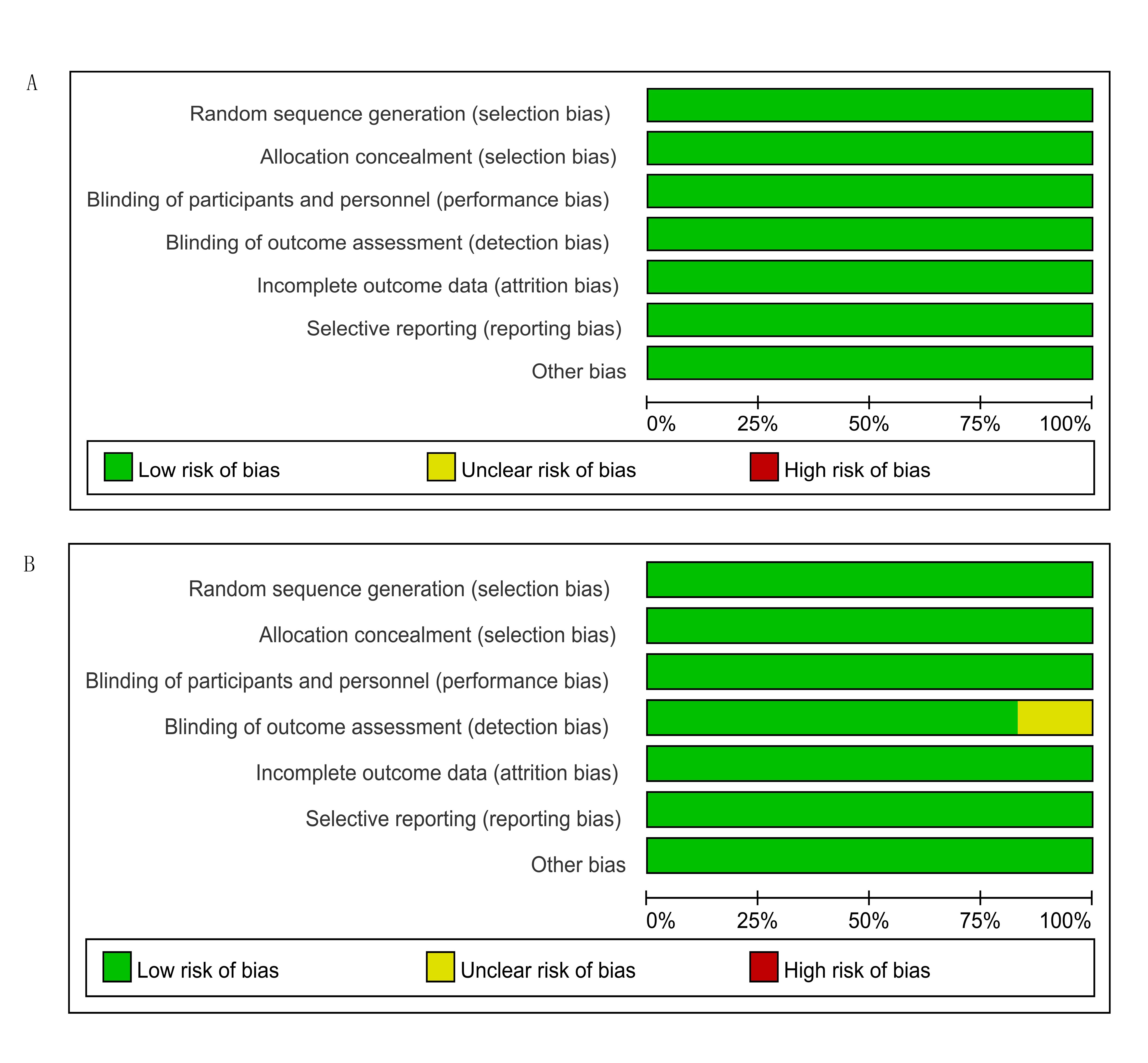

Supplement: Supplementary file 5 [file Image1.TIF]

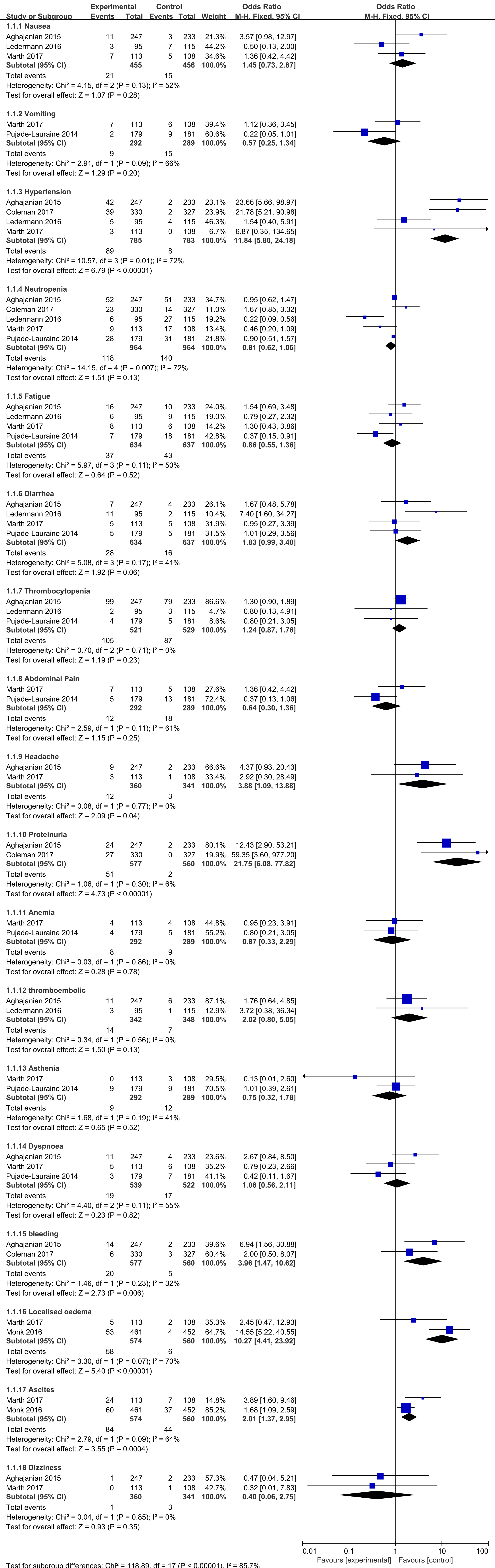

Supplement: Supplementary file 6 [file Image7.TIF]

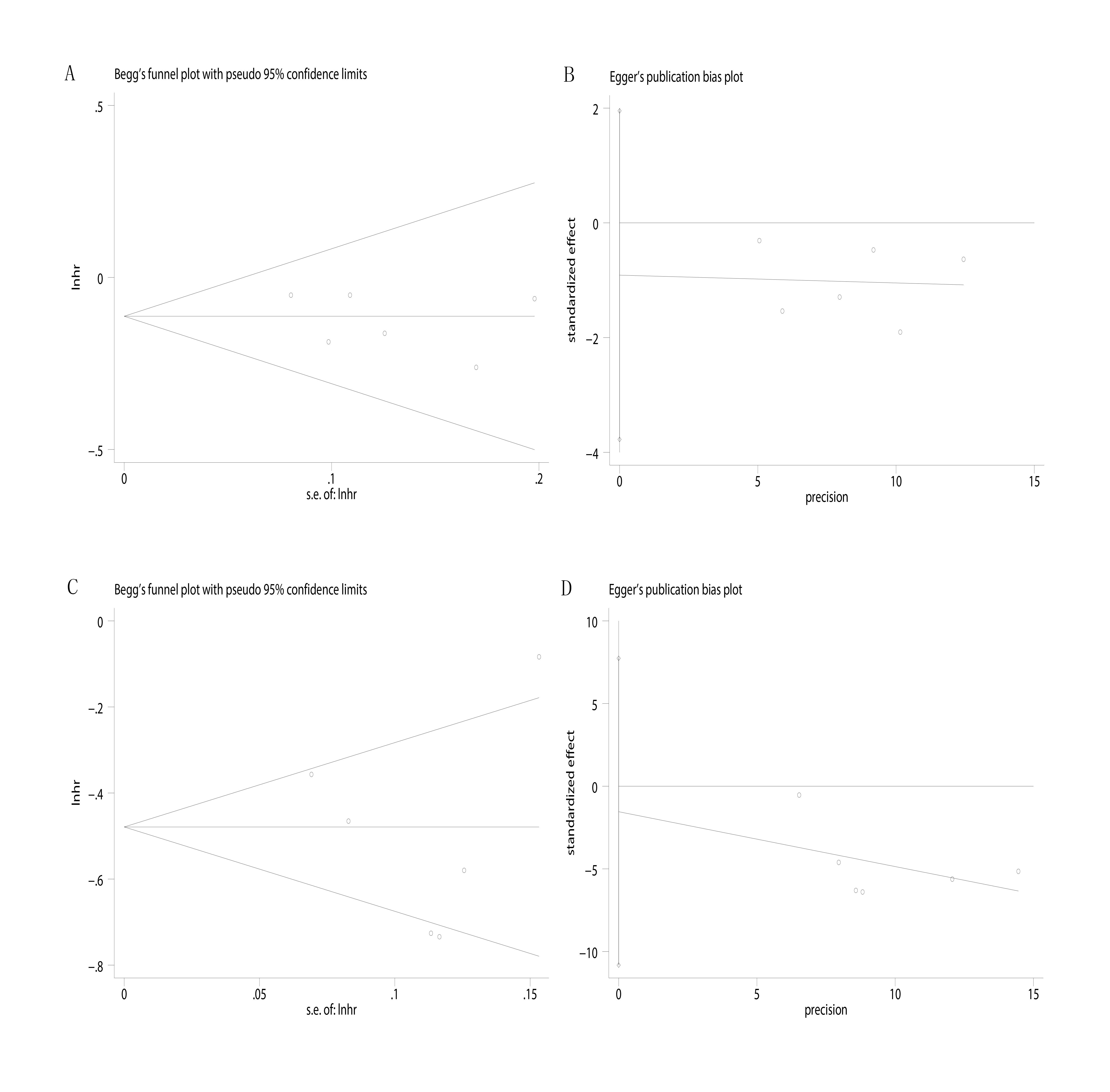

Supplement: Supplementary file 7 [file Image5.TIF]
